# Development and Validation of an Artificial Intelligence-Based Motion Analysis System for Upper Extremity Rehabilitation Exercises in Patients with Spinal Cord Injury: A Randomized Controlled Trial

**DOI:** 10.3390/healthcare12010007

**Published:** 2023-12-19

**Authors:** Hyun Jong Lee, Seung Mo Jin, Seck Jin Kim, Jea Hak Kim, Hogene Kim, EunKyung Bae, Sun Kook Yoo, Jung Hwan Kim

**Affiliations:** 1 Department of Clinical Rehabilitation Research, Rehabilitation Research Institute, National Rehabilitation Center, Seoul 01022, Republic of Korea; ileehunjongi@naver.com (H.J.L.); hogenek@umich.edu (H.K.); 2 Department of Rehabilitation Exercise, National Rehabilitation Center, Seoul 01022, Republic of Korea; 3 Ministry of Health and Welfare, Sejong 30113, Republic of Korea; 4 REMO Inc., Seoul 06159, Republic of Korea; 5 Department of Medical Engineering, Yonsei University College of Medicine, Seoul 03722, Republic of Korea

**Keywords:** artificial intelligence, motion feedback system, spinal cord injury, rehabilitation

## Abstract

In this study, we developed an AI-based real-time motion feedback system for patients with spinal cord injury (SCI) during rehabilitation, aiming to enhance their interest and motivation. The effectiveness of the system in improving upper-limb muscle strength during the Thera band exercises was evaluated. The motion analysis program, including exercise repetition counts and calorie consumption, was developed using MediaPipe, focusing on three key motions (chest press, shoulder press, and arm curl) for upper extremity exercises. The participants with SCI were randomly assigned to the experimental group (EG = 4) or control group (CG = 5), engaging in 1 h sessions three times a week for 8 weeks. Muscle strength tests (chest press, shoulder press, lat pull-down, and arm curl) were performed before and after exercises. Although both groups did not show significant differences, the EG group exhibited increased strength in all measured variables, whereas the CG group showed constant or reduced results. Consequently, the computer program-based system developed in this study could be effective in muscle strengthening. Furthermore, these findings may serve as a valuable foundation for future AI-driven rehabilitation exercise systems.

## 1. Introduction

Spinal cord injury (SCI) is a condition where injury to the central nervous system causes the loss of various physical functions. As patients with SCI experience numerous constraints due to reduced function of different areas, including the sensory, motor, and autonomic nervous systems, a variety of rehabilitation treatments are required to ensure independent daily activities to enhance quality of life [1,2,3]. Upper extremity functions are essential in mobility-associated activities in patients with SCI, such as the pushing of a wheelchair. Therefore, the preservation or enhancement of upper extremity functions is an important goal in rehabilitation [4].

Previous studies have reported that physical functions, such as muscle strength or range of motion, can be improved via resistance training, including Thera-band exercises, for the recovery of upper extremity functions in patients with SCI [5,6]. Hicks et al. showed that upper extremity exercise programs could enhance patients’ quality of life while increasing upper extremity muscle strength [1,2,3]. To strengthen the upper-extremity muscles, elastic resistance training is required. Examples of elastic resistance training include weight-lifting exercises using a dumbbell or a weight machine, or those applying the elasticity of rubber using a Thera-band [7].

Thera-band exercises are widely used in the rehabilitation of patients with SCI, in addition to their use by the general population, as they assist with improving muscle strength and endurance as well as balance and functional movements. Thera-band exercises are effective in increasing the accuracy of motion by strengthening the damaged muscle and increasing the coordination and range of motion of joints. In addition, such exercises allow for varying levels of difficulty and methods so that a suitable exercise can be selectively used on each patient according to their condition [8].

While Thera-band exercises can be performed readily at home, there is no way to ascertain whether the motion being followed is appropriate as the exercises are mostly performed without an instructor’s guidance. Hence, if one continues to exercise in an inaccurate way, there could be a risk of injury, and the efficiency of the exercise may be reduced [9].

To resolve this problem, studies have suggested applying a device, such as a mirror, that can provide visual feedback to enable one to check their posture during exercise [10,11,12,13,14]. Moreover, recent studies have investigated the use of real-time motion analysis systems to provide the user with instant feedback on their motion [13,15,16]. In conventional motion analysis, a reflection marker or an inertial measurement unit sensor is attached to the participant’s body for wired or wireless data collection to conduct motion analysis. However, this method is highly inconvenient for the user, as setting up the device poses numerous difficulties [17].

As an alternative, marker-less motion analysis systems with devices such as the Kinect camera have been developed. The recent advancement of artificial intelligence (AI) technology has enabled real-time motion analysis solely using a webcam or a mobile phone camera [18,19,20,21]. Despite technological advancements, only a few studies have explored the use of AI technology in the rehabilitation exercises of subjects with SCI.

Therefore, this study developed an AI-based real-time motion feedback system with a camera and applied it to subjects with SCI performing Thera-band exercises. The aim was to verify the effectiveness of the system on the improvements in muscle strength using the foundation of upper extremity functions.

## 2. Materials and Methods

### 2.1. Study Design

**Figure 1:**
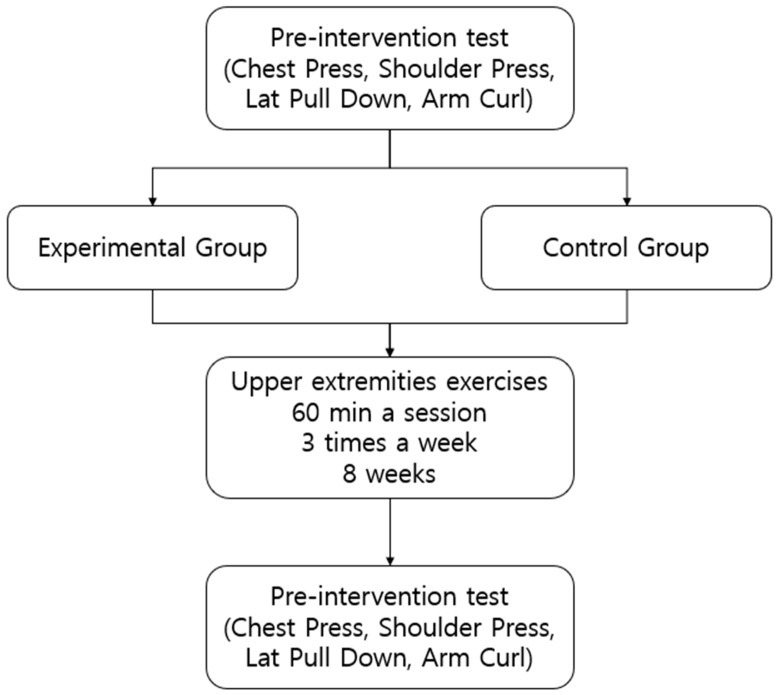
The experimental protocol.

This study was a single-blinded, randomized controlled trial (Registration code: PRE20230727-005). All participants at pre-intervention selected an envelope without knowing which group they would belong to and were randomized into either the Experimental Group (EG), performing exercises with the novel program, or the Control Group (CG), performing general exercises without the computer program developed in this study. As shown in Figure 1, the EG and the CG performed band exercises (1 h/session; three sessions/week) for 8 weeks. If a participant missed a session due to personal reasons during the intervention period, they were instructed to perform the same exercise at home using the video recording of the day. A physical fitness test (chest press, shoulder press, lat pull-down, arm curl) was performed before and after the intervention.

The two groups were distinguished as follows: the EG performed the exercise by watching the instructor as well as the screen in front of them, and the CG performed the exercise by watching the instructor only. A single instructor led the exercise session at different times for the two groups, while all participants in a group performed the exercise together with the single instructor rather than in a 1:1 session. This study was approved by the Institutional Review Board of the National Rehabilitation Center (IRB No. NRC-2022-04-029).

### 2.2. Participants

The participants of this study were subjects with chronic SCI > 6 months, aged between 49 and 80 years, who could understand the instructions and independently perform the exercise with their status on the American Spinal Injury Association impairment scale, A–D [22]. The clinical trial was conducted over two months, from October 1 to November 30. Those unable to perform the upper-extremity exercises independently and those with cardiovascular or musculoskeletal disorders were excluded.

### 2.3. Physical Fitness Test Devices

**Figure 2:**
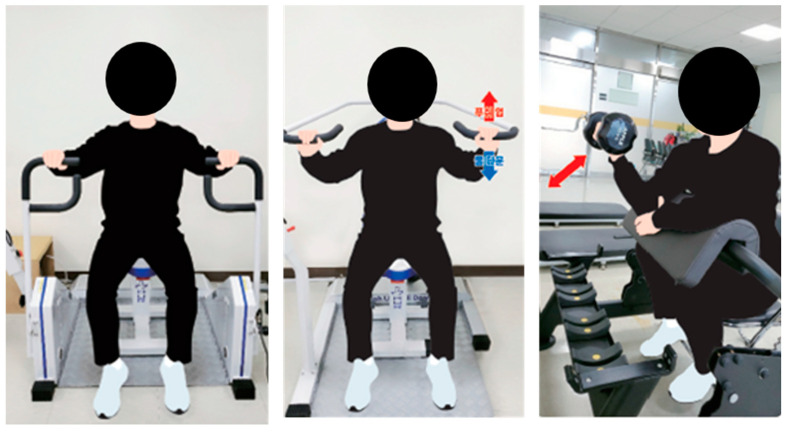
The muscle strength test ((**left**): chest press, (**middle**): shoulder press, lat pull down, and (**right**): arm curl). Korean to English translation of the right column: From the top: 푸시업 (Push up), 풀다운 (Pull down).

The muscle strength test was performed, as shown in Figure 2, as a pre- and post-intervention test. A chest press device and a shoulder press device were produced by Hur (Hur Limited, Kokkola, Finland). PR1, a strength measurement device produced by Hur and attached to the Hur chest press device and the Hur shoulder press, was also used to measure muscle strength for 5 s. PR1 is a tensile-compressive load cell used to measure muscle strength using the isometric method. It is attached to each device to measure the target variable only during a test with an isometric protocol. The data were collected in kg, which could be converted to Nm per the producer’s manual [23].

For the chest press (right and left) and shoulder press (shoulder press and lat pull-down), we took two measurements each and used the best value. For the arm curl, the number of lifts of the set weight was counted for 1 min, and the final score was determined by multiplying the set weight by the number of lifts.

### 2.4. AI-Based Motion Analysis and Visual Feedback Program

The AI-based motion analysis program for the upper extremity exercises in this study was designed using MediaPipe, developed by Google (Google Inc, Mountain View, CA, USA). MediaPipe automatically detects the 33 human joint points, and as it is light software, the program can be run using only the central processing unit without the graphics processing unit. MediaPipe was also used in a study by Latreche et al. to achieve a 95% agreement with the goniometer result by physical therapists in terms of reliability and validity. Therefore, it was determined to be suitable for use in developing the program in this study [20,21].

**Figure 3:**
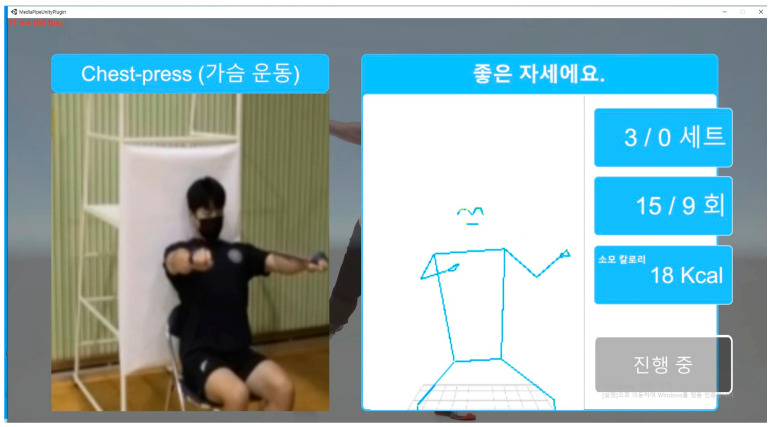
Motion analysis system display. Korean to English translation of the right column: From the top: 좋은 자세예요 (Good Posture!), 세트 (Set), 회 (Times), 소모 칼로리 12 Kcal (Calories consumed: 12 kcal), and 진행 중 (In progress).

The novel program hardware system in this study had three components: a 20-inch monitor, a mini-PC (AMD Ryzen 5, 5600 G with Radeon Graphics 3.90 GHz), and a webcam. The webcam was installed on the front to film the participant, in the form of a mirror, and to analyze their motion. The monitor had a split screen to display the model posture and the results of motion analysis on one side and the posture of the user on the other side (Figure 3).

The real-time motion analysis provided visual feedback on the user’s motion and was consequentially used to motivate the user to continue to exercise. The program included the functions of motion repetition counting and calorie calculation based on the motion repetition.

### 2.5. Motion Repetition Counter

In reference to previous studies [24,25,26], where upper extremity exercises using a Thera-band were used on people with SCI, the exercise protocol to activate the crucial upper extremity muscles, from the shoulder depressors to shoulder extensors, shoulder external rotators, scapular retractors, and triceps muscles, was revised every 1 or 2 weeks and was accommodated as required to individually suit the fitness purpose of this study. Furthermore, it allowed the motion analysis system to count the repetitions of the three motions: chest press, shoulder press, and arm curl.

All images were created using Unity. A single count with four values (x, y, z, and w) was recorded on the user’s motion outside the set boundary, as shown in Table 1, after which the counter was reset. In Unity, the values x, y, z, and w represent orientation and rotation with quaternion values. Here, x, y, and z denote vector values, representing each axis, while w is a scalar value indicating the magnitude of the rotation. These quaternion values were employed to achieve smooth 3D motion. 

### 2.6. Calorie Calculation

An equation was developed to calculate the calorie consumption of each motion during exercise. Due to the lack of data on the metabolic equivalent (MET) values for band exercises in patients with SCI, the MET values of a band exercise for the upper extremities were developed for the general population in a study by Michael et al., and the resistance values per band color reported in the study by Uchida et al. were applied [27,28,29].

As shown in Table 2, the MET values per band color were induced, and in reference to a study by Eileen G.C. on oxygen consumption by patients with SCI (1 MET = 2.7 mL/kg/min, not 3.5 mL/kg/min), the calorie calculation during exercise was formulated according to the discussions among the researchers of this study [30].

The body weights of the patients with SCI were applied with the MET values per band color (Table 2), and the unit oxygen consumption per minute (resting = 2.7 mL/kg/min) in patients with SCI to the calorie consumption equation per session. The exercise time of 0.33 min was applied based on the assumption that three sessions can be performed for 1 min.

**Figure 4:**
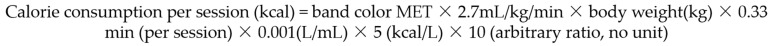
The pragmatic equation of calorie consumption per session. Abbreviations: MET (metabolic equivalent values, no unit).

Additionally, as approximately 5 kcal is generated at 1 L of oxygen consumption, the result was multiplied by 0.001 (to convert L into mL) and 5 (kcal/L). In the process of calculating the calories of a single measurement, the result was multiplied with a 10-fold coefficient after discussions by the research team so as to reflect the recovery from muscle damage, increase the level of scientific evidence, and show the user a numerical value of calorie consumption that could elicit interest in exercise and motivation. The participants were notified before exercising that the calculated calorie consumption would not be an accurate experimental value. The equation of calorie consumption per session is shown in Figure 4.

### 2.7. Exercise Outcome

The program developed in this study allowed the input of body weight and band color before exercise at the respective predetermined entries.

**Figure 5:**
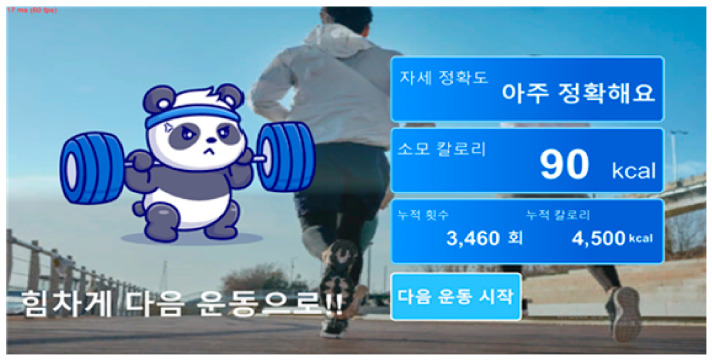
The session ending (result summary) display. Korean to English translation of the right column: From the top of the right column 자세 정확도 (Postural accuracy), 아주 정확해요 (Very accurate!), 소모 칼로리 (Consumed calorie), 누적횟수 3460 회 (Cumulative repetition: 3460 times), 누적 칼로리 4500 kcal (Cumulative calorie: 4500 kcal), 다음 운동 시작 (Start the next set) and at left column 힘차게 다음 운동으로!! (With this energy, let’s move on to the next!).

Upon the completion of an exercise session, a result table was displayed (for example, Figure 5) for the EG subjects. The data, presented next to a character design, included the accuracy of the arbitrary exercise posture feedback (not discussed in this paper), the calorie consumption, and the calculations of cumulative repetition and calories of all sets of the exercise performed.

### 2.8. Exercise Interventions for the EG and CG

Both the EG and CG performed a 60 min exercise session consisting of a 10 min warm-up, 40 min of main exercise, and a 10 min cool-down. In the 40 min of main exercise, the participants performed upper extremity exercises using a band and/or a dumbbell under an instructor’s guidance. The instructor led identical upper-extremity exercises for the EG and the CG without exercise quantity or instruction variation.

The upper extremity exercises comprised motions using a band to increase the range of motion of the shoulder joint and to train the large muscles of the back and chest, as well as the relatively small muscles, such as the biceps and triceps. To prevent adaptation to exercise, the exercise intensity was set to increase a step at a time in 1 or 2-week intervals. To ensure a gradual change in exercise intensity, the band color was altered or motions using a dumbbell were added between the band exercises. The band color at the start for the basic exercises was recommended as the green color, while the color was changed between yellow and black depending on the exercise capacity of the user.

**Figure 6:**
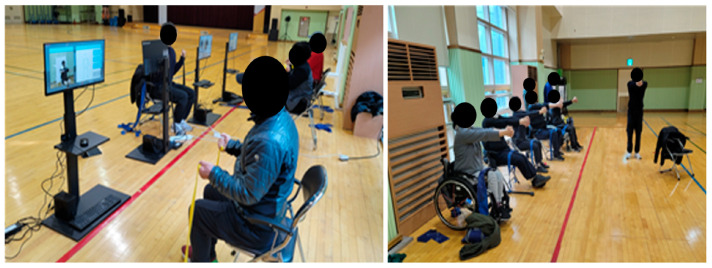
The Experimental Group (EG) is on the left, and the Control Group (CG) is on the right.

Both the EG and the CG faced the same instructor and followed the motions guided by the instructor. The EG watched their motions on the computer program monitor developed in this study, which was set in front of them, and asked for the instructor’s further guidance continuously when necessary. The CG watched the instructor only, without the computer program (Figure 6). While the program used by the EG allowed the real-time counting of the repetition of the three motions (chest press, shoulder press, and arm curl), it did not have the ability to count other motions whose repetition count was not displayed on the monitor. The participants thus exercised, solely watching their reflection on the screen, which acted like a mirror.

After 8 weeks of exercise, a usability test was conducted on the EG and the instructor to review the benefits and drawbacks, room-for-improvement suggestions, and general opinions of program use. The usability test was a questionnaire containing 10 structured items, rated on a 5-point Likert scale, and 5 semi-structured items. Each free-form interview to record any other opinions was also conducted [31].

Regarding the 5-point scale questionnaire, the scores showed: 1 point, complete rejection; 2 points, rejection; 3 points, normal; 4 points, agreement; and 5 points, full agreement was achieved.

### 2.9. Statistical Analysis

The results obtained from the pre- and post-intervention physical fitness tests in the EG and CG were compared. The Kolmogorov–Smirnov test was used to verify data normality. Regarding the comparison between the groups, an independent *t*-test was performed for post-values separately from pre-values within each group, while within-group comparisons were performed using paired *t*-tests to compare pre- and post-test values. Among the variables of the participants’ general characteristics, continuous variables were expressed as means and standard deviations, and categorical variables were expressed as frequencies and percentages. All data analyses were performed using SPSS 26.0 version for Windows (IBM Corp., Armonk, NY, USA), and the level of significance was set at a *p*-value of 0.05.

## 3. Results

For the 8-week intervention period, the two groups participated in a total of 24 sessions of exercise. Nine participants were enrolled and randomized into two groups: the EG (n = 4) and the CG (n = 5). The EG had three males and one female; two had a cervical injury, one had a thoracic injury, and one had a lumbar injury. The CG had two males and three females; one had a cervical injury, three had a thoracic injury, and one had a lumbar injury (Table 3). Table 4 and Table 5 present the exercise outcomes. The results demonstrate an increase in strength across all tested items in the EG but a constant, reduced/increased level in the CG.

In the EG, all measured variables increased for all participants. A1, in particular, showed an increase in the chest press from 13.72 kg to 24.29 kg on the left side and from 10.67 kg to 19.75 kg on the right side. Likewise, A3 showed a notable increase from 14.64 kg to 31.17 kg on the left side and from 12.89 kg to 36.54 kg on the right side. A4 exhibited the largest increase in the shoulder press, from 20.87 kg to 55.14 kg. For all tested items, an average increase of 10% was observed in A2, the oldest participant in this study.

Regarding the CG, the overall level of increase was low compared with the EG, and despite an overall increase, several items were found to have decreased. B3, in particular, showed a reduced level across all items except the lat pull-down and arm curl. Additionally, B1 and B2 showed a reduced level for the lat pulldown. With the exception of B4, all participants showed reduced levels for the shoulder press. B4 was the only participant exhibiting an increase across all tested items in the CG.

All participants in both groups showed an increase in the Arm Curl test.

To examine the differences in the exercise effect between the EG and the CG, an independent *t*-test was performed on the pre- and post-intervention tests. To determine the intra-group exercise effectiveness with time, the results of the pre- and post-intervention tests were compared (Table 6). For all tested items, the pre- and post-intervention test effects did not vary significantly between the EG and the CG. This suggests that the effects of exercise in the two groups were similar statistically.

Comparing the pre- and post-intervention test results in each group to determine the exercise effect indicated a significant difference for the bilateral arm curl in both the EG and CG. This suggests that the effects of exercise in the two groups were similar statistically. However, the CG showed a significant difference for the right chest press.

**Figure 7:**
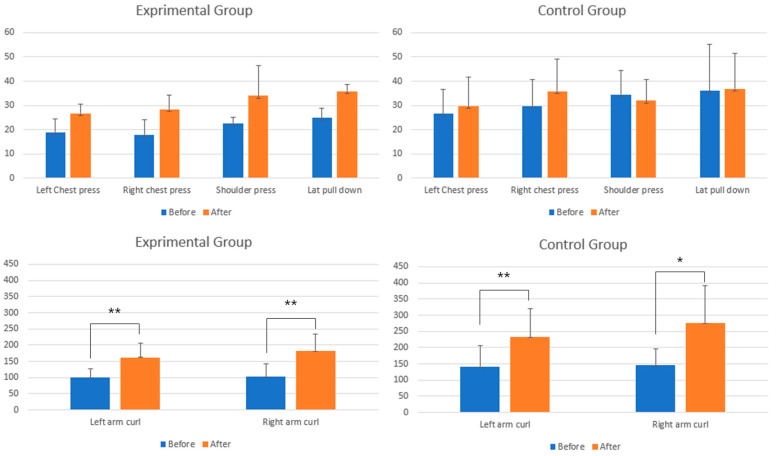
Comparison of pre- and post-intervention test means by group (* *p* < 0.05, ** *p* < 0.01).

Figure 7 was produced to examine the trend change in each tested motion. In the EG, on average, all tested items showed an increase of more than or equal to 40% after exercise, as follows: left chest press (42%), right chest press (58%), shoulder press (50%), lat pull down (44%), left arm curl (64%), and right arm curl (76%). However, in the CG, all tested items except the arm curl showed a negligible level of increase or a reduced level as follows: left chest press (16%), right chest press (24%), shoulder press (−4%) (regarded as decreased), lat pull down (1%) (regarded as constant), left arm curl (68%), and right arm curl 80%.

The usability test on the system developed in this study, involving one instructor and all four participants in the EG, revealed that the usability score of the watching-monitor frequency of use was 5 by the user and 4 by the instructor. For the overall program ease of use, the score was 5 by both the user and the instructor, implying that they uniformly regarded the new program as easy to use. However, for the methods of use, the usability score was 3 for the user and 5 for the instructor. For satisfaction of use, the usability score was 5 according to the user and 3 according to the instructor. The usability score for completeness was 2 for both the user and the instructor, implying a degree of dissatisfaction.

For the review of program use, most of the users stated that they liked being able to see themselves through real-time motion analysis and that the instructor guided them on how to perform the exercise. The drawback was the low diversity of the exercise program types. Most users pointed out the following improvements: the need for a greater variety of exercise program types and system stabilization to allow them to exercise independently at home, at their convenience, without an instructor.

Regarding the instructor, the system itself was good as it was not complex and easy to use; however, he suggested a number of points that could be improved, especially in terms of the number of exercise program types, the design of the visual feedback program, and the non-existence of negative feedback.

## 4. Discussion

An AI-based motion analysis system was developed in this study, and the usability and effectiveness of the system for muscle strengthening were evaluated. The results revealed no significant difference between the two groups; however, all tested items showed an improvement in the EG, the users of the computer-aided exercise system, while a constant or reduced level (non-improvement) was observed in the CG, the control exercise without a computer aid.

The biggest issue might have been the insufficient number of participants. The same instructor guided both groups through an identical exercise session. However, the participants in the EG group, having the ability to observe themselves through the motion analysis system during exercise, were presumed to experience increased motivation due to the visual feedback. In contrast, the participants in the CG only watched the instructor during the exercise; therefore, it is possible that they performed the exercise in inaccurate postures in the absence of 1:1 direct guidance. This could have resulted in reduced exercise effectiveness, and the lack of feedback on postural correction could have decreased participant motivation, reducing the effect. Only B4 in the CG exhibited an increase across all tested items, presumably because she could effectively understand the instructor and independently perform the exercises in accurate postures as she had the best physical state with a lumbar injury. Our results are similar to those of a study by Baptista et al., where visual feedback was provided to people without SCI, and of a study by Sayenko et al., where the effect of visual feedback on patients with SCI was investigated [14,16].

In the present study, the four participants in the EG included three males and one female; two had a cervical injury, one had a thoracic injury, and one had a lumbar injury. The participants in the CG included two males and three females; one had a cervical injury, three had a thoracic injury, and one had a lumbar injury. This indicated a slightly higher proportion of cervical injuries, a relatively more severe condition, in the EG. The mean age of the participants was 68 years in the EG and 59 years in the CG, indicating an age gap of approximately 10 years. Nevertheless, while the exercise effect is generally known to be lower in older adults and severely affected people, the results of this study showed a trend of a quantitatively higher effect in the EG (with higher ages and more severe conditions), which implies that a difference in age and severity did not suppress the effectiveness of the intervention.

The motion analysis system used by the participants in the EG was developed using the MediaPipe algorithm, which is light and rapid in processing, as the system had to provide real-time feedback on participant motions. The animation effect was removed as much as possible, and as an all-in-one, stand-type device was used, the webcam had to be positioned in line with the center of the user. The use of AI technology allowed the two-dimensional image to be analyzed in three dimensions; however, the accuracy of motion detection decreased in the chest press with front-to-back anterior movements. In a follow-up study, an angular zone of motion analysis should be set so that the camera can be installed above and diagonally to the user to increase the accuracy of motion analysis.

The usability test involving the users and the instructor indicated a satisfactory result. The users stated that it was good to watch themselves during exercise, allowing them to recognize their inaccurate postures. The instructor was satisfied that he could guide a number of individuals, not only one at a time, smoothly through the use of the computer system. Both the subjects in the EG and the instructor stated that the system was easy to use; however, there was room for improvement regarding system completeness.

This study has several limitations. As both EG and CG comprised patients with SCI, not people without physical disability, it was difficult to recruit participants for this clinical study due to the COVID-19 pandemic. Consequently, the low number of participants may have resulted in a decrease in the statistical power and study closure, not reaching intended recruitment. External factors could not be controlled because all participants were chronic free-living outpatients (not inpatients). Therefore, it is difficult to attribute the results in the two groups purely to the use or non-use of these study sessions. Additionally, while efforts were made to involve all participants in all sessions throughout the 8-week intervention period, some participants had to miss one session or more due to illness or personal reasons. Although such participants were instructed to perform the exercise at home using the video recording of the day, this is a factor that could not be controlled by the investigator.

## 5. Conclusions

As this study only included a small number of subjects with SCI, the results cannot be generalized to all patients with various SCI statuses. Nonetheless, the significance of this study lies in effectively verifying the potential use of an AI-based real-time motion analysis system in rehabilitation exercises through this single-blind, randomized controlled trial.

Furthermore, while the computer program developed in this study consisted of only three motions, it is noteworthy that it included the fundamental upper extremity exercises for individuals with disabilities and that the future goal of the program was to enhance extended basic physical fitness, allowing the users to subsequently perform other exercises. The analysis of the effectiveness of the exercise did not reveal a significant difference between the EG and the CG; however, the trend of a markedly definite effect was observed for the EG in the actual clinical strength assessment. This suggests that this novel computer-aided program is effective, and we intend to verify the results of the study through further experiments in the future. This study suggests the cornerstone insight that visual feedback may be beneficial and fundamental for the development of AI-based rehabilitation exercise programs in the future.

## Figures and Tables

**Table 1:** Counting and reset criteria for each motion.

	Chest Press	Shoulder Press	Arm Curl
Counting	x, z, and w of Left_elbow: x < 0, z > 0, w > 0.5	y and w of Left_elbow: y < 0, w > 0	y of Left_wrist > y of Left_elbow
Reset	x, z, and w of Left_elbow: x > 0.5, z < 0.5, w < 0	y and w of Left_elbow y > 0, w < 0	y of Left_wrist < y of Left_elbow

**Table 2:** MET for each band color.

Band Color	Yellow	Red	Green	Blue	Black
MET	2.77	2.88	2.93	3.1	3.29

Abbreviations: MET (metabolic equivalent values, no unit).

**Table 3:** Participant characteristics.

Code	Age	Sex	Weight (kg)	Height (cm)	Onset Year	ASIA Score	Neurologic Level of Injury	Types of Spinal Cord Injuries	Number of Exercise Sessions Participated
A1	66	F	70.1	145.1	1982	D	Lumbar	Incomplete	20
A2	80	M	65.3	157.7	2014	D	Cervical	Incomplete	23
A3	56	M	72.7	165.6	2020	D	Cervical	Incomplete	24
A4	70	M	102.4	181.4	2007	C	Thoracic	Incomplete	22
B1	64	M	70.8	170	2019	D	Thoracic	Incomplete	24
B2	49	F	75.2	162	2017	A	Thoracic	Complete	19
B3	59	F	43.3	153	2018	C	Cervical	Incomplete	22
B4	57	F	58.4	159.6	2020	D	Lumbar	Incomplete	23
B5	68	M	84.6	170	2019	D	Thoracic	Incomplete	24

Code numbers with A represent subjects in the Experimental Group (EG), while those with B represent subjects in the Control Group (CG). Abbreviations: ASIA, American Spinal Injury Association; F, female; M, male.

**Table 4:** Exercise outcome (strength, kg) in the Experimental Group (EG).

	Left Chest Press	Right Chest Press	Shoulder Press	Lat Pull Down	Left Arm Curl	Right Arm Curl
A1						
Pre-intervention test	13.72	10.67	22.08	19.86	54	54
Post-intervention test	24.29	19.75	23.78	38.69	90	120
A2						
Pre-intervention test	28.05	25.79	26.79	28.3	100	96
Post-intervention test	29.27	29.35	30.25	31.64	180	152
A3						
Pre-intervention test	14.64	12.89	20.72	21.84	114	99
Post-intervention test	31.17	36.54	26.94	37.77	183	198
A4						
Pre-intervention test	18.95	22.35	20.87	29.21	130	165
Post-intervention test	22.21	27.65	55.14	34.96	198	258

**Table 5:** Exercise outcome (kg) in the Control Group (CG).

	Left Chest Press	Right Chest Press	Shoulder Press	Lat Pull Down	Left Arm Curl	Right Arm Curl
B1						
Pre-intervention test	29.01	37.88	39.6	59.55	175	185
Post-intervention test	40.17	50.32	39.52	54.02	240	360
B2						
Pre-intervention test	23.8	32.58	34.9	42.03	148	160
Post-intervention test	28.5	37.73	31.11	34.28	260	300
B3						
Pre-intervention test	17.3	18.21	20.72	6.84	69	108
Post-intervention test	14.38	17.62	16.4	16.9	112	120
B4						
Pre-intervention test	18.18	16.14	27.47	21.76	78	76
Post-intervention test	19.49	23.86	31.17	25.87	177	177
B5						
Pre-intervention test	44.64	44.04	49.67	49.67	240	205
Post-intervention test	46.06	49.69	41.17	53.1	372	432

**Table 6:** Inter- and intra-group comparisons (n = 9).

	Experimental Group (n = 4)	Control Group (n = 5)	Inter- Group Differences	*p* ^†^	ŋ^2^
Left chest press (kg)					
Pre-intervention test	18.84 ± 6.54	26.58 ± 11.14	4.33 (−12.08; 20.75)	0.463	0.190
Post-intervention test	26.73 ± 4.18	29.72 ± 13.39			
Intra-group changes	−7.89 ± 7.01 (−19.06; 3.27)	−3.13 ± 5.23 (−9.63; 3.37)			
*p* ^‡^	0.11	0.252			
Right chest press (kg)					
Pre-intervention test	17.92 ± 7.28	29.77 ± 12.21	4.21 (−17.05; 25.48)	0.573	0.117
Post-intervention test	28.32 ± 6.89	35.84 ± 14.83			
Intra-group changes	−10.39 ± 9.13 (−24.92; 4.13)	−6.07 ± 4.7 (−11.92; −0.22)			
*p* ^‡^	0.107	0.045 *			
Shoulder press (kg)					
Pre-intervention test	22.61 ± 2.84	34.47 ± 11.13	12.53 (−7.44; 32.51)	0.140	0.571
Post-intervention test	34.02 ± 14.32	31.87 ± 9.81			
Intra-group changes	−11.41 ± 15.35 (−35.84; 13.01)	2.59 ± 4.61 (−3.13; 8.32)			
*p* ^‡^	0.234	0.277			
Lat pull down (kg)					
Pre-intervention test	24.8 ± 4.64	35.97 ± 21.39	10.74 (−4.96; 26.44)	0.118	0.612
Post-intervention test	35.76 ± 3.17	36.83 ± 16.46			
Intra-group changes	−10.96 ± 7.56 (−23.00; 1.08)	−0.86 ± 7.36 (−10.00; 8.27)			
*p* ^‡^	0.063	0.806			
Left arm curl (score)					
Pre-intervention test	99.5 ± 32.71	142.0 ± 70.98	−16.50 (−61.62; 28.62)	0.329	0.311
Post-intervention test	162.75 ± 49.13	232.2 ± 97.3			
Intra-group changes	−63.25 ± 18.96 (−93.42; −33.07)	−90.2 ± 35.92 (−134.80; −45.59)			
*p* ^‡^	0.007 **	0.005 **			
Right arm curl (score)					
Pre-intervention test	103.5 ± 45.85	146.8 ± 53.7	−27.00 (−168.11; 114.11)	0.586	0.110
Post-intervention test	182.0 ± 59.93	276.6 ± 129.73			
Intra-group changes	−78.5 ± 20.76 (−111.53; −45.46)	−129.8 ± 81.67 (−231.21; −28.38)			
*p* ^‡^	0.005 **	0.024 *			

Data are shown as means ± standard errors and means (95% confidence intervals) for intra- and inter-group changes (* *p* < 0.05, ** *p* < 0.01). ^†^: independent *t*-test was performed on the difference values between pre- and post-measurements in both the experimental and control groups. ^‡^: paired *t*-tests were performed separately for pre- and post-values within each group.

## Data Availability

The data presented in this study are available on request from the corresponding authors.
